# Surface Morphology and Optical Properties of Hafnium Oxide Thin Films Produced by Magnetron Sputtering

**DOI:** 10.3390/ma16155331

**Published:** 2023-07-29

**Authors:** José de Jesús Araiza, Leo Álvarez-Fraga, Raúl Gago, Olga Sánchez

**Affiliations:** 1 Instituto de Ciencia de Materiales de Madrid, Consejo Superior de Investigaciones Científicas, Cantoblanco, 28049 Madrid, Spain; araiza@fisica.uaz.edu.mx (J.d.J.A.); leo.alvarez@icmm.csic.es (L.Á.-F.); rgago@icmm.csic.es (R.G.); 2 Unidad Académica de Física, Universidad Autónoma de Zacatecas, Calzada Solidaridad Esq, Paseo La Bufa, Fracc, Progreso, Zacatecas 98060, Mexico

**Keywords:** HfO_2_, magnetron sputtering, bonding structure, morphology, blister, optical properties

## Abstract

Hafnium oxide films were deposited on sapphire and silicon (100) substrates using the DC reactive magnetron sputtering technique from a pure hafnium target at different discharge power levels. The influence of the cathode power on the chemical composition, morphology, crystallographic structure and optical properties of the films was investigated. X-ray diffraction (XRD), energy dispersive X-ray analysis (EDX) and Fourier-transform infrared spectroscopy (FTIR) were employed to determine the chemical composition and bonding structure. In all cases, the films were found to be amorphous or nanocrystalline with increased crystalline content as the sputtering power was increased, according to XRD and FTIR. In addition, EDX showed that the films were oxygen-rich. The effect of power deposition on the surface topography and morphology of the films was studied using atomic force microscopy (AFM) and scanning electron microscopy (SEM). The AFM and SEM images revealed the emergence of mound morphologies as the cathode power was increased. These features are related to blistering effects probably due to the presence of stress and its promotion within the film thickness. Finally, the optical properties showed an average transmission of 80% in the visible range, and the refractive index determined by spectral ellipsometry (SE) was found to be in the range of 1.85–1.92, close to the reported bulk value. SE was also used to study the film porosity observed by SEM, which can be related to the oxygen-rich character of the films.

## 1. Introduction

Hafnium oxide (HfO_2_) is a highly intriguing chemical compound in scientific research due to its unique electrical and dielectric properties such as high dielectric constant (~25), low density of interface states with silicon and high band gap (5.8 eV) [1]. It is a high-k dielectric material that is widely used as a gate oxide in complementary metal–oxide–semiconductor (CMOS) technology to enhance the performance of microelectronic devices [2], being a very promising candidate for replacing silicon oxide in metal–oxide–semiconductor (MOS) devices. In addition, HfO_2_ thin films have been found to exhibit excellent optical properties such as a high refractive index and low absorption, making them ideal for the use in optical coatings and waveguides [3]. HfO_2_ is also a highly refractory material with excellent thermal stability and mechanical properties [4]. Due to these properties, HfO_2_ has emerged as a promising candidate for a wide range of industrial applications, including its use as a catalyst [5]. The applications of HfO_2_ as a promising catalyst include the conversion of biomass-derived feedstocks, production of hydrogen through water splitting and the removal of pollutants from wastewater [6]. On the other side, HfO_2_ has interesting structural properties too [7] that motivated its previous studies as a ferroelectric material. In particular, it presents monoclinic, orthorhombic and tetragonal phases, and, at least, its orthorhombic structure induces a remnant polarization that brings its ferroelectric behavior [8,9]. Consequently, HfO_2_ thin films have also shown promising applications in various fields, including memory devices, sensors and energy storage systems. For instance, HfO_2_-based memory devices have been reported to exhibit excellent performance characteristics such as high speed, low power consumption and high reliability. HfO_2_ thin films have also been studied for their potential use as solid-state electrolytes in lithium-ion batteries, where they can offer higher energy density and better safety conditions as compared to conventional liquid electrolytes [10].

In general, physical, optical and electrical properties of HfO_2_ films are highly dependent on their microstructure, morphology and chemical stoichiometry, where these aspects depend on particular deposition processes and post-deposition treatments. The growth of HfO_2_ thin films can be achieved using various methods including, among others, chemical [11] and physical [12] vapor deposition, atomic layer deposition (ALD) [13] and the sol–gel process [14]. Each method has its advantages and limitations, and the choice of a particular method depends on the specific requirements of the aimed application. For example, ALD is a widely used method for the deposition of high-quality HfO_2_ thin films due to its excellent conformity and precise thickness control. As reported by Liao et al. [15], ALD produces fine-grained films (grain sizes of a few tens of nanometers) with a monoclinic (*m*-HfO_2_) crystalline structure. However, the ALD technique is limited to very thin films (few tens of nanometers) and, in addition, may not be suitable for large-scale production. Alternatively, HfO_2_ thin films grown by a sputtering technique have gained considerable attention due to its potential advantages over other deposition methods such as scalability and high throughput. Sputtering is a physical vapor deposition technique that utilizes high-energy ions to dislodge atoms from a target material and deposit them onto a substrate. Some of the advantages of growing the HfO_2_ thin films using sputtering are high purity, good adhesion, controllable thickness and uniformity. The control of the variables of the sputtering process makes it possible to tailor the obtained properties for the desired application. Changes in growth variables can induce and control lateral and depth uniformity, giving rise to superficial and bulky structures that can modify optical properties [16]. In particular, these characteristics can be affected by the energy of the incident species, interaction with the environment (pressure and sputtering or reactive gases), temperature and substrate surface condition. Moreover, sputtering deposition is widely used for thin film growth due to its ability to coat large surface areas, making it easy to scale-up industrially. In most of the works in which growth by sputtering is reported, RF sputtering from HfO_2_ cathodes and annealing post-treatments at high temperatures are typically used [4,16]. On the contrary, the deposition of HfO_2_ thin films by DC reactive magnetron sputtering using a pure metallic hafnium target are scarce. The latter allows for the control of the elemental composition of the resulting films in a broader range by properly adjusting the process parameters. Power deposition is a determinant parameter to modify the internal and surface structure of the films in a controlled way according to the desired characteristics [17].

In this work, HfO_2_ thin films with a high refractive index, broadband transparency and low spectral absorption were deposited by DC reactive magnetron sputtering at room temperature on single-crystal silicon and sapphire substrates, without post-annealing treatments. We established a correlation between the chemical composition, crystalline structure and surface morphology and the optical properties (refractive index, *n*, and optical band gap, *E_g_*) of the films. Despite the fact that the deposition process was carried at room temperature without subsequent annealing treatments, the films showed excellent optical properties. 

## 2. Materials and Methods

HfO_2_ films were deposited at room temperature on p-(100)-oriented silicon and hexagonal *α*-Al_2_O_3_ (0001) substrates by DC reactive magnetron sputtering from a highly pure metallic hafnium (99.99%) 3-inch target. Silicon substrate used is p-type, and prior to the deposition, the substrates were cleaned in successive ultrasonic baths of trichloroethylene, acetone, ethanol and distillate water for 10 min each to degrease and eliminate any foreign materials on their surfaces. Finally, the substrates were dried under nitrogen flow. After pre-sputtering for five minutes, the deposition process was carried out at a constant pressure of 2.5 × 10^−3^ mbar for 40 min with a gas mixture of Ar and O_2_ which was adjusted by individual mass flow controllers (Ar/O_2_ = 9/3 sccm). The power applied to the Hf cathode (W_Hf_) was varied at 100, 150 and 200 W, the distance between the target and the substrate was set at 15 cm in all depositions, and no external heating or sample rotation was used. The base pressure was around 3.6 × 10^−6^ mbar before the deposition process. 

The crystalline structure of the deposited samples was determined using X-ray diffraction (XRD) (Bruker 10 system with Cu-kα radiation in Bragg–Brentano *θ*-2*θ* geometry). The diffraction patterns were collected with 0.05° steps and an acquisition time of 1 s/step. The morphology of the samples was analyzed with a field emission scanning electron microscope (FE-SEM) (Verios-460) operated at 20 kV. This microscope is equipped with an energy dispersive X-ray (EDX) spectrometer. The EDX spectra were registered over three different surface regions of each film, and the mean at.% compositions were calculated. The compositional analysis was supported by Rutherford backscattering spectrometry (RBS) measurements at the 5 MV HVEE Tandetron accelerator located at the Centro de Micro-Análisis de Materiales of the Universidad Autónoma de Madrid. RBS experiments were performed with ^4^He^+^ projectiles for a dose of 10 µC with an energy of 1.8 MeV. The backscattered particles were detected with a silicon detector located at a scattering angle of 170°. The chemical composition of the samples was extracted using the SIMNRA 7.07 simulation software [18]. Atomic force microscopy (AFM) images were collected with a Nano-Observer equipment from Scientec (France) in tapping mode to measure surface topography and to analyze the roughness of the films. The images were processed and analyzed with the open-source software Gwyddion [19]. The optical properties were determined based on the direct optical transmission measurements carried out in the wavelength range of 190–900 nm using a Shimadzu SolidSpec-3700 spectrophotometer. The bonding structure was studied by Fourier transform infrared spectroscopy (FTIR) using an ABB MB 3000 spectrophotometer working in transmission mode in the MIR region (400–4000 cm^−1^). Spectroscopic ellipsometry (SE) was performed by utilizing a GES-5E ellipsometer from SOPRA, covering a spectral range from 200 to 900 nm (1.4–6 eV). The SE curves were fitted with the WinElli II (SOPRA©) 2.2 software with a Cauchy dispersion model, which is appropriate to describe the dielectric function of transparent materials in the measured spectral range [20]. The thickness of the films obtained by SE was also checked by a Veeco Dektak 150 mechanical profilometer. 

## 3. Results and Discussion

Deposition parameters, deposition rate and chemical composition of the samples determined by EDX are shown in Table 1. Samples are named HfO-x, where x refers to the deposition power. It is clear that as the power applied to the hafnium target increased, the deposition rate also increased. This trend was accompanied by a decrease (increase) in oxygen (hafnium) content in the films according to the EDX results. The EDX values were cross-checked by measuring RBS in the HfO-200 sample and obtaining exactly the same composition. In any case, oxygen content much higher than the stoichiometric value (67 at.%) was detected in the films. The progressive decrease in O_2_ content with the increase in W_Hf_ can be related to the nature of the reactive process, since the growth conditions move towards the metallic mode of the target as W_Hf_ is increased (reduced target poisoning). 

**Figure 1:**
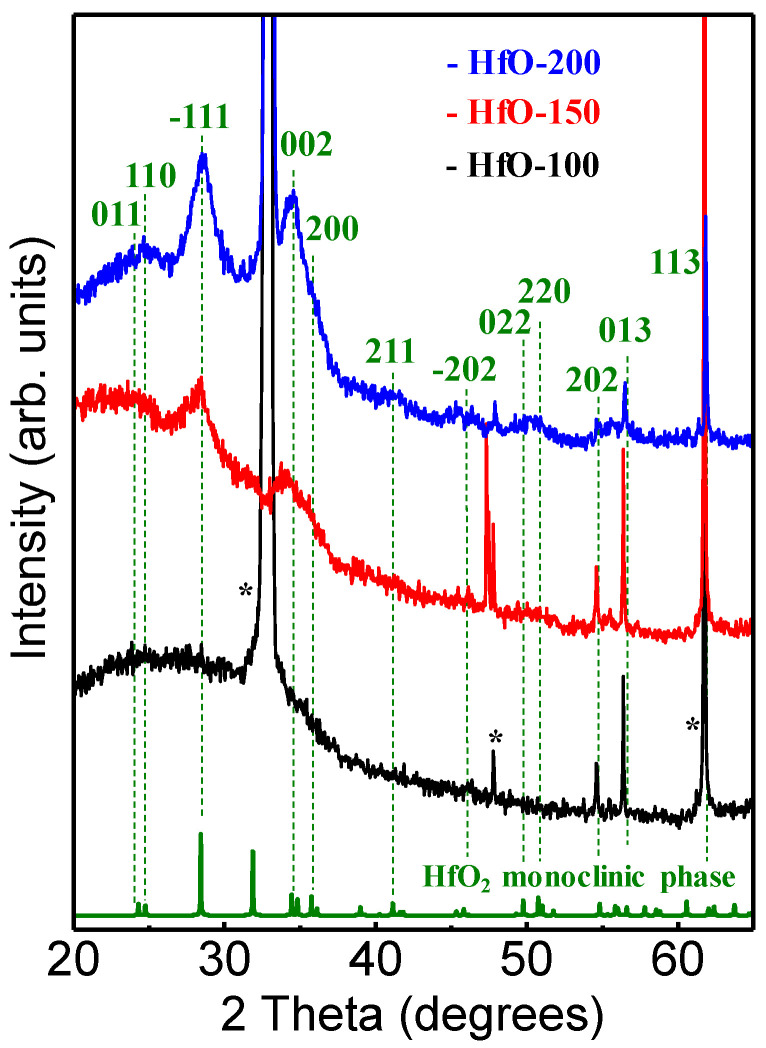
XRD measurements for HfO-x films at different deposition powers. Peaks marked with an asterisk (*) are attributed to the XRD reflections of the silicon substrate. The reference powder diffraction pattern from monoclinic HfO_2_ is also shown (*JCPDS 43-1017*).

The XRD patterns recorded as a function of the power applied to the hafnium target are shown in Figure 1. The diffractograms were compared with the powder diffraction pattern (PDF) of *m*-HfO_2_ available in the database (*JCPDS 43-1017*), and all the peaks, including the minor ones were indexed. At W_Hf_ = 100 W, a very broad band along 2θ = 30–40° indicates the X-ray amorphous character of the sample. Nevertheless, the existence of precursor seeds of different crystalline hafnium oxides is not discarded, since some of them present Bragg reflections in the *2θ* range swept by this band [4]. Three very small peaks at higher *2θ* values can be related to the crystalline HfO_2_ phase according to the reference reflections of HfO_2_ also shown in Figure 1. As W_Hf_ increased, it became possible to detect specific crystalline reflections related to the HfO_2_ formation. The main peaks were located around 28.4 and 34.5°, corresponding to the principal planes observed for the *m*-HfO_2_ phase structures in ($\bar{1}$11) and (002) planes, respectively [21]. At W_Hf_ = 200 W, the signals from (002) and ($\bar{1}$11) orientations were clearly detected, but the relatively broad width of the peaks indicated the nanocrystalline nature of the films. The presence of several reflections indicated that different out-of-plane grain orientations took place, without any preferential texture. In addition, the overlap of different reflections might have occurred in the 31–34° range. Therefore, the data show that the reactive magnetron sputtering process produced disordered HfO_2_ films, with a structure evolving from X-ray amorphous at a low DC power towards nanocrystalline *m*-HfO_2_ at higher powers. It should also be noted that the increase in the thickness with the increase in the applied power may also amplify this trend. Similar results were reported with different deposition techniques and with post-annealing treatment in a controlled atmosphere [22,23].

**Figure 2:**
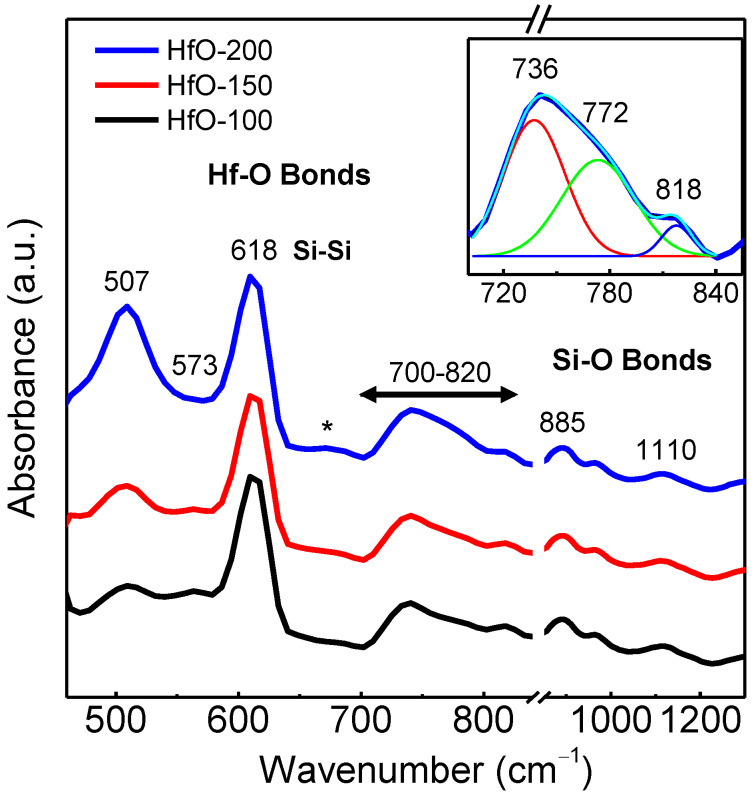
Absorption IR spectra of HfO-x films prepared at different deposition powers. Inset shows the deconvolution in the 700 to 820 cm^−1^ range for HfO-200 film.

Figure 2 shows the FTIR spectra of the deposited films. The observed bands can be ascribed to some of the reported typical IR vibrational modes known for amorphous (*a*-HfO_2_) and *m*-HfO_2_ phases [16,23,24]. The bands related to the presence of Hf-O bonds were reported at different zones for the middle IR region within 400–800 cm^−1^ [16,23] associated with the vibrational modes (named A_u_ and B_u_) for *m*-HfO_2_. To study the bands in the IR spectra in detail, we considered three zones where absorption peaks were detected as discussed below. First, we performed the deconvolution of the broad band in the region of 462–640 cm^−1^, where three peaks around 507, 573 and 618 cm^−1^ were obtained. A feature around 507 cm^−1^ is clearly observed in Figure 2 and can be attributed to A_u_ and B_u_ vibrational modes in *m*-HfO_2_. These modes are related to atomic displacements parallel to the c-axis of the unit-cell and the atomic displacement parallel to the *x*-axis of the unit-cell, respectively [24]. The signal from 570 cm^−1^ can be attributed to amorphous Hf-O bonds [16]. Note that the amorphous character of these films was confirmed by the XRD measurements. The signal around 618 cm^−1^ can be considered a Si-Si vibration [25] coming from the substrate. The second zone considered was related to a very weak signal in the 641–671 cm^−1^ range (marked with *), and it was also related to the overlapped signal of A_u_ and B_u_ modes of the *m*-HfO_2_ structure. In this case, they account for the displacement already considered from the c- and *x*-axis at 641 and 651 cm^−1^ values, respectively [24]. However, in all the spectra, the signal was very weak, and only qualitative trends could be extracted. Finally, deconvolution in the 700 to 820 cm^−1^ range showed three bands (see inset in Figure 2): two of them, at 736 and 772 cm^−1^, were associated with a phonon mode of *m*-HfO_2_ [23] and another band, at 818 cm^−1^, was related to the Si-O bond. The band at 736 cm^−1^ is considered a B_u_ vibrational mode [24], while the band at 772 cm^−1^ in this work coincides with the reported value and remains almost constant in all the IR spectra [23]. The deconvoluted peak can be itself divided into two components, a signal coming from the interfacial SiO_2_ for symmetrical stretching and another from the B_u_ vibrational mode parallel to the *x*-axis, which is relatively more intense in the aforementioned report [24].

Some IR peaks (507, 618 and 651 cm^−1^) in this work were displaced to higher wave-numbers with respect to the reference spectrum of HfO_2_, which is related to higher force constants. In the spectra, signals attributed to silicon phonons (618 cm^−1^) from the silicon substrate and small peaks or broad bands at 818, 885 and 1110 cm^−1^, related to Si-O bonds from the substrate–film interface, were also detected [26,27]. In particular, the 885 cm^−1^ peak was observed for core silicon non-bridged with oxygen [27] for a relatively low partial pressure of oxygen. Table 2 shows the position of different important Hf-O bands in the spectra, together with their corresponding areas and the phases they are related to. These areas are an estimation of the contribution to the spectra of the different phases in the films [28]. The spectra were normalized with respect to thickness, allowing us to establish that an increase in power deposition leads to an increase in the number of Hf-O bonds present in the films. The data in Table 2 suggest that the content of the Hf-O bonds associated with the *a*-HfO_2_ phase slightly decreased with the increase in the deposition power, while the crystalline phase clearly increased. In all cases, the structure of the HfO-x films appeared to be composed of an amorphous matrix with a certain nanocrystalline component. The increase in the deposition power led to an enhancement of the crystalline content in the films, in agreement with the results obtained by XRD.

**Figure 3:**
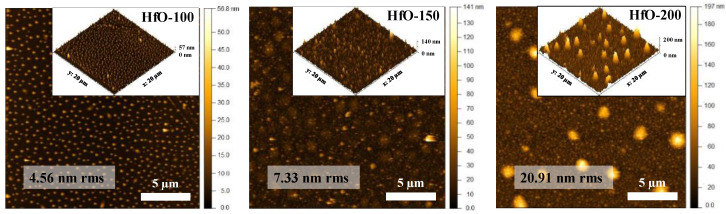
AFM topography images indicating the (rms) roughness and the corresponding 3D AFM images with a scan area of 20 × 20 µm^2^ for HfO-x films’ growth at different deposition powers.

The influence of W_Hf_ on the film surface morphology is illustrated using AFM (Figure 3). The surface roughness increased from around 5 to 21 nm for the HfO-100 and HfO-200 films, respectively. At low W_Hf_, the surface topography is rather homogeneous, and the obtained roughness is assumed to be a characteristic feature of the whole sampled surface. At high W_Hf_, the situation is complex, since the contribution to the roughness comes from two different features: first, relatively high hill-like structures (mounds) and a smoother granular structure in the global plane. The grain morphology is also present on the surface of the hill-like structures, indicating the same nature and structure of the material giving origin to both morphological features. These phenomena were observed previously in the case of crystal growing by ALD [22], but in that case, the structure was related to higher temperatures that favor smoother surfaces with a higher crystalline structure. Nevertheless, in both cases, there was a smaller and relatively general granular structure convoluted with the formation of large mounds [29].

It was observed that the local roughness related to the granular morphology at a deposition power of 100 W is similar to that in the films produced at a higher W_Hf_. However, the isolated mound morphology changed drastically with the increase in the applied power (thickness). First, the morphology presented a high density of narrow dots with a height of few nm at 100 W. At higher power (thickness), there appeared mounds that were relatively broader and taller (few tens of nanometers) with a more disperse distribution and, consequently, a larger interdistance (see, for example, the image of the HfO-200 sample). Note the image contrast at 150 W, where the intermediate situation is presented showing the large incipient structures superimposed with the smaller and shallower initial mounds. From the AFM images, we can estimate the height, average distance and surface coverage (density) of mound morphologies as displayed in Table 3. For the estimation of the distance between the mounds, the values were obtained from the radial power spectral density (PSD) derived from the crossover in the slope change of the PSD curve as described in the literature [30]. In this case, the calculation was obtained from 10 × 10 µm^2^ AFM micrographs. The results from the characteristic scales obtained are shown in Table 3. The analysis indicated that the size of and distance between the grains raised with the increase in W_Hf_. It has been previously reported that surface defects resulting in the formation of such type of mounds are due to the effects that come more from energy contributions [31], although a possible thickness effect cannot be ruled out in this case. In general, it can be seen that W_Hf_ has an important effect on the dimensions of the mounds (height, projected area and volume) and distances between them.

**Figure 4:**
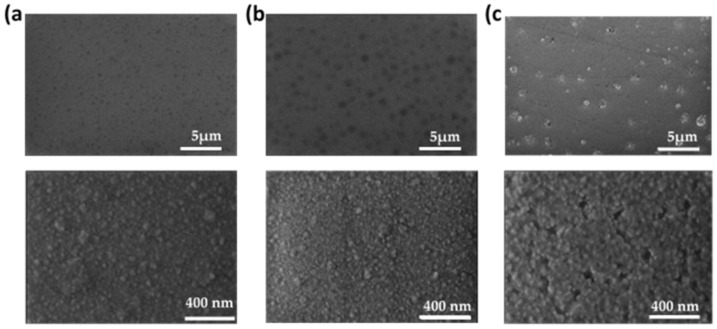
(**a**) Top view SEM images of HfO film deposited at 100 W (**a**), 150 W (**b**) and 200 W (**c**) with 50,000× (**upper row**) and 150,000× (**lower row**) magnifications.

In Figure 4, the SEM images of the samples deposited at different powers are shown. SEM analysis supports the observed morphologies by AFM, with the tendency of forming isolated mounds, as evidenced by the image contrast. As the power increased, the definition of the mounds was enhanced, with a clear influence in the height, projected area and volume and distances between them. This effect is especially clear in the case of sample HfO-200. The higher-magnification images (bottom row) also evidence the similar background granular morphology in all the cases and the formation of open pores at the highest W_Hf_. 

**Figure 5:**
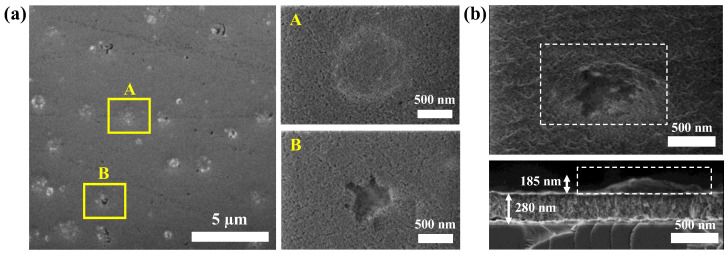
(**a**) Top-view SEM images of HfO-200 film: (**A**,**B**) regions are the zoom-in images of the yellow squares (2.5 µm × 1.75 µm), respectively. (**b**) Tilted-view and cross-section SEM images of HfO-200 thin film a with visible collapsed mound and porous structure.

Figure 5 shows different top view SEM images and cross-section SEM images for the HfO-200 thin film. From the cross-section SEM micrograph, the porous structure in the film microstructure could be observed in addition to the formation of a mound on the background surface. In this way, the height of the mound (~185 nm) can be compared with the average values obtained from the AFM analysis in Table 3. In contrast to AFM, SEM showed an intriguing effect of mound collapse or, more specifically, cracking of the large islands (see Figure 5a). The latter can be seen with more detail in the high-magnification images of the surface (panels A and B). A close up look at the surface using SEM in Figure 4b, reveals that the local structure of the domes is formed by grains and voids on the surface, with a similar structure to that in the flatter regions. 

**Figure 6:**
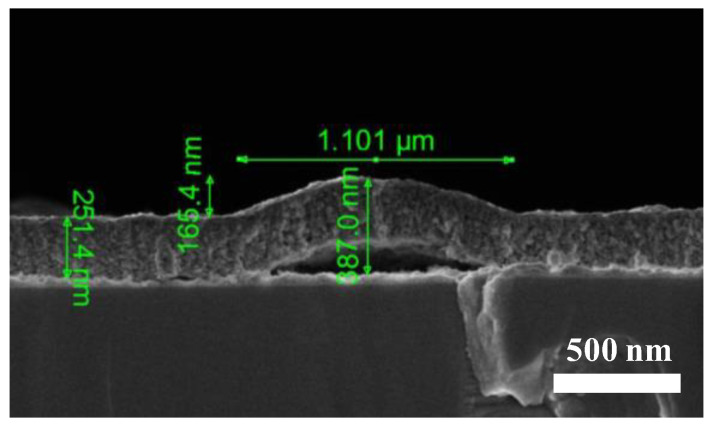
Cross-sectional SEM image depicting a detailed view of a blister formation on a HfO-200 thin film.

The formation of protruding structures at W_Hf_ = 200 W and the formation of empty voids underneath (as evidenced by SEM) resemble the blistering effect observed in thin films. It should be noted that this phenomenon was already observed in HfO_2_ grown by the sol–gel technique but was less noticeable than in this work [32]. In addition, the diameter of the observed structures was much larger than the film thickness, a condition required for blistering [33]. The presence of blistering was clearly confirmed in the SEM images shown in Figure 6. Note that the dimensions of the mounds (height and diameter) are in agreement with the values obtained by the AFM analysis reported in Table 3. 

There are two potential mechanisms that could cause the blistering effect: stress and gas trapping [34,35,36]. Both mechanisms are not exclusive, and, frequently, it is very difficult to determine the dominant factor. Blistering effects of thin films have been normally reported upon post-growth annealing treatments and/or direct ion irradiation [37,38]. However, in our case, this effect was observed in *as-deposited* films. Moreover, despite the large O_2_ content, there is no evidence of significant bubble formation by gas trapping. Therefore, the most plausible scenario could be the local failure of the film adhesion (dewetting) at the surface interface caused by the emergence of compressive stress [33]. The release of strain energy would then result in the buckling process giving rise to the formation of mounds and voids underneath them. The stress increase with the increase in W_Hf_ could then be related to the larger film thickness and/or the higher deposition rate. It should be noted that this hypothesis is supported by the SEM images. 

**Figure 7:**
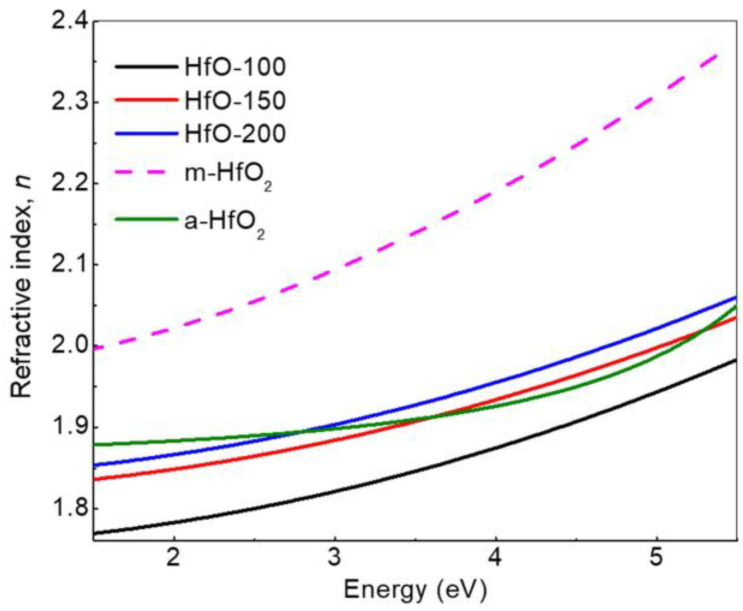
Refractive index *n* extracted from the SE fitting for HfO_2_ films deposited at different W_Hf_ levels and comparison with reported studies for *a*-HfO_2_ [3] and *m*-HfO_2_ [39].

From ellipsometric parameters Ψ and Δ, obtained by SE, simulations were carried out that allowed us to obtain the value of the thickness and the refractive index for the films grown at different deposition powers. To carry out the calculations, a basic structure formed by the crystalline silicon substrate with a native SiO_2_ on top and a dispersion model for the main HfO_2_ film (Si-cr/SiO_2_/HfO_2_) were used. In addition, an outer layer with an effective medium approximation (EMA) of film and voids (air) was included to account for film roughness. The optical constants of the Si substrate and SiO_2_ were provided by the WinElli II software. In the case of the HfO_2_ layer, the Cauchy dispersion model for transparent film was used to fit the optical response. The goodness of the fitting was assessed by the coefficient of determination (R2) values of the regression analysis, which were always above 0.9. The thickness values calculated by SE are comparable to those obtained by profilometry (79, 138 and 297 nm for HfO-100, HfO-150 and HfO-200, respectively). The typical expressions of the fitting model for the refractive index and the extinction coefficient of the HfO_2_ layer read as follows: *n* = *A* + *B/λ*^2^ *+ Cλ*^4^, and *k* = 0 for the spectral region considered (240–890 nm). From the simulation, the refractive indices obtained for the films in the measured spectral region are shown in Figure 7. The values for A and B are shown in Table 4. These values are close for the parameter A reported for *m*-HfO_2_ [39,40] and *a*-HfO_2_ [3], which are 1.9627 and 1.85 in the transparent region (where the value of the absorption coefficient is taken as 0), respectively. In the model, the constant C was considered 0. As can be seen in Figure 7, the refractive index of these films is greater than or equivalent to that obtained for *a*-HfO_2_ in other reported works [3]. For low energies, the refractive index values were in the range of 1.77–1.85, and in the range of 1.98–2.06 for near-UV. These values are very appropriate for thin layer applications in various electronic devices [16] and optical systems [41]. It should be noted that the presence of mounds on the surface does not seem to affect the optical properties. This can be understood from the formation of continuous films with homogeneous thickness together with buckled regions (mounds).

In order to gain further information about the film microstructure, a second optical model was used for the SE fitting by using the reported optical properties of *m*-HfO_2_ from the database of the WinElli II software. In this case, the model considered the main film as an EMA with a mixture of voids and *m*-HfO_2_. This model provided a similar fitting goodness as compared to that obtained with the Cauchy dispersion law. However, in this case, we were able to make an estimate of the porosity from the calculated concentration of voids in the samples. The values obtained were 21, 15 and 12% for HfO-100, HfO-150 and HfO-200, respectively. These values correlate with the oxygen excess extracted from EDX. In addition, the density of the HfO-200 sample determined by the combination of RBS data and film thickness provides a value around 9.18 g/cm^3^. From this value, we can estimate a 5.16% porosity by comparison with the bulk density value of HfO_2_ reported in the literature [42]. Therefore, the high content of oxygen can be related to the porous condition of the films, as can be seen in Figure 5 and Table 4.

**Figure 8:**
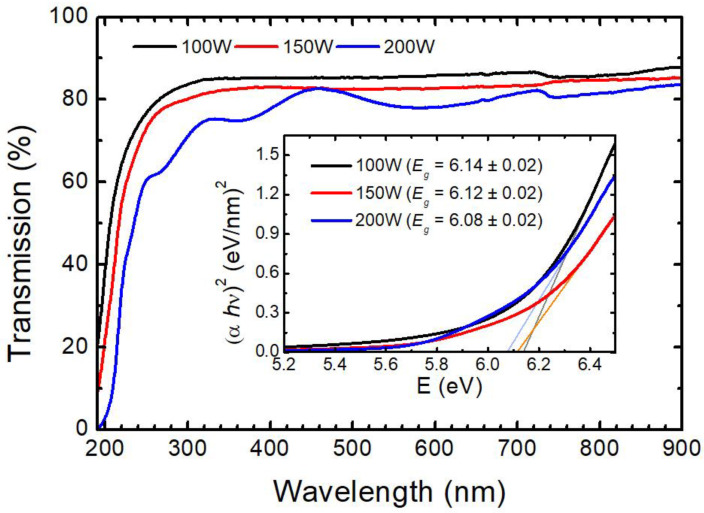
Optical transmission of HfO-x films deposited on *α*-sapphire at different deposition powers. Inset: Band gap energy calculated according to Tauc expression from optical characterization.

The optical transmission spectroscopy measurements in the UV–Vis–near-IR range are shown in Figure 8. The average optical transmission is over 80% in the whole range between 300 and 900 nm. The variation in the value of the optical gap energy for the studied power deposition is indicated in the inset of the figure. The bandgap was calculated using the Tauc plot considering direct transitions [43]. This assumption was taken into account by the almost amorphous nature of the samples [44]. The optical band gap, *E_g_*, was determined from the expression *αhv~(hv-E_g_)^r^*, where *α* is the optical absorption coefficient, *h* is the Planck’s constant, *v* is the frequency of radiation, and the exponent *r* depends on the nature of optical transition. In the present case, the best fit in the Tauc plot was obtained for *r*= 1/2, which shows that direct transitions in these films are allowed. The calculations showed that the energy of the optical gap decreased as the deposition power used in the growth process increased, leading to high values in all cases, considering that the value usually reported for *m*-HfO_2_ is 5.8 eV [45].

## 4. Conclusions

This study was carried out to investigate the surface morphology, microstructure and optical properties of hafnium oxide films deposited by reactive magnetron sputtering as a function of the deposition power. In all cases, the HfO-x films were rich in oxygen and, their structure appeared to be composed of an amorphous matrix with some content of nanocrystalline *m*-HfO_2_. The content of nanocrystalline *m*-HfO_2_ increased with the discharge power. The resulting surface morphology, as determined by AFM and SEM, showed the emergence of relatively large mounds that grew larger with the power applied to the hafnium target. These features coexisted with a more general and smaller granular structure that was related to the structural nature of the nanocrystalline HfO_2_. On the contrary, the mound formation was related to blistering effects where partial dewetting took place, leading to film buckling and the formation of protruding features. The origin of such a process could be related to the stress fields generated during the growth, which were more intense for larger film thicknesses. It is important to note that, despite the fact that the deposition process was carried at room temperature without subsequent annealing treatments, the films showed excellent optical properties. In particular, they presented an average transmission of 80% in the visible range, a refractive index in the range of 1.85–1.92 and optical band gaps over 6.0 eV.

## Figures and Tables

**Table 1:** Deposition conditions and atomic composition of HfO_2_ films determined by EDX.

Sample	W_Hf_	Thickness (nm ± 10%)	Deposition Rate (nm/min)	Hf (at.%)	O_2_ (at.%)
HfO-100	100	80	2.0 ± 0.2	16 ± 2	84 ± 2
HfO-150	150	150	3.8 ± 0.4	20 ± 2	80 ± 2
HfO-200	200	300	7.5 ± 0.8	26 ± 2	74 ± 2

**Table 2:** Contributions to the IR bands of the HfO-x films.

Sample	507 cm^−1^ *m*-Hf-O	570 cm^−1^ *a*-Hf-O	700–820 cm^−1^ Region			Area	
			736 cm^−1^ *m*-Hf-O	772 cm^−1^ *m*-Hf-O	818 cm^−1^ Si-O	(arb. Units)	
						Crystalline	Total
HfO-100	2.27	1.01	0.91	1.23	-	4.41	5.85
HfO-150	2.70	0.84	1.03	0.84	-	4.57	5.91
HfO-200	5.20	0.83	1.27	1.06	-	7.53	8.50

*m*-Hf-O and *a*-Hf-O are abbreviated terms for monoclinic Hf-O and amorphous Hf-O, respectively.

**Table 3:** Characteristic morphological features of the mound structures extracted from the AFM images.

Sample	Average Distance (nm)	Height (nm)	Average Diameter (nm)	Projected Area (µm^2^)	Volume (µm^3^)
HfO-100	650 ± 130	27 ± 6	261 ± 68	0.1038 ± 0.04	1.703 × 10^−3^
HfO-150	1380 ± 275	62 ± 12	866 ± 246	0.7523 ± 0.56	1.634 × 10^−2^
HfO-200	2650 ± 500	176 ± 36	1183 ± 357	1.6490 ± 0.48	1.629 × 10^−1^

**Table 4:** Cauchy parameters calculated for optical properties of HfO-x films’ growth at different deposition powers.

Sample	Parameter A	Parameter B	% Voids *
HfO-100	1.7520	0.01189	21
HfO-150	1.8200	0.01107	15
HfO-200	1.8366	0.01151	12
*m*-HfO_2_	1.9627 *	0.02200	-
*a*-HfO_2_	1.8500	0.01170	-

* From the calculated values for Cauchy model for dielectric function in the large wavelength region.

## Data Availability

The data presented in this study are available in this article.
